# A Prognostic Signature Based on Immunogenomic Profiling Offers Guidance for Esophageal Squamous Cell Cancer Treatment

**DOI:** 10.3389/fonc.2021.603634

**Published:** 2021-02-24

**Authors:** Jianyao Gao, Ting Tang, Baohui Zhang, Guang Li

**Affiliations:** ^1^ Department of Radiation Oncology, The First Affiliated Hospital of China Medical University, Shenyang, China; ^2^ Department of Physiology, School of Life Science, China Medical University, Shenyang, China

**Keywords:** esophageal squamous cell carcinoma, prognosis, signature, immunotherapy, cancer drug resistance, tumor microenvironment

## Abstract

Our study aimed to develop an immune prognostic signature that could provide accurate guidance for the treatment of esophageal squamous cell cancer (ESCC). By implementing Single-Sample Gene Set Enrichment Analysis (ssGSEA), we established two ESCC subtypes (Immunity High and Immunity Low) in GSE53625 based on immune-genomic profiling of twenty-nine immune signature. We verified the reliability and reproducibility of this classification in the TCGA database. Immunity High could respond optimally to immunotherapy due to higher expression of immune checkpoints, including PD1, PDL1, CTLA4, and CD80. We used WGCNA analysis to explore the underlying regulatory mechanism of the Immunity High group. We further identified differentially expressed immune-related genes (CCR5, TSPAN2) in GSE53625 and constructed an independent two-gene prognostic signature we internally validated through calibration plots. We established that high-risk ESCC patients had worse overall survival (P=0.002, HR=2.03). Besides, high-risk ESCC patients had elevated levels of infiltrating follicle-helper T cells, naïve B cells, and macrophages as well as had overexpressed levels of some immune checkpoints, including B3H7, CTLA4, CD83, OX40L, and GEM. Moreover, through analyzing the Genomics of Drug Sensitivity in Cancer (GDSC) database, the high-risk group demonstrated drug resistance to some chemotherapy and targeted drugs such as paclitaxel, gefitinib, erlotinib, and lapatinib. Furthermore, we established a robust nomogram model to predict the clinical outcome in ESCC patients. Altogether, our proposed immune prognostic signature constitutes a clinically potential biomarker that will aid in evaluating ESCC outcomes and promote personalized treatment.

## Introduction

Esophageal carcinoma (EC) is one of the most common cancers worldwide and the 6^th^ leading cause of cancer related deaths globally (1). Esophageal squamous cell cancer (ESCC) is the commonest subtype of esophageal carcinoma. ESCC incidence is particularly high in East Asia, where it accounts for accounting about 90% of ECs (2). Despite recent advances in diagnosis and treatment, most ESCC cases are diagnosed at an advanced stage and are characterized by rates of metastasis and relapse (3, 4). As a result of high genetic heterogeneity and development of drug resistance, ESCC prognosis remains very poor, with a 5-year survival rate of <30% (5, 6). Recent research has aimed to uncover biomarkers with early diagnostic and prognostic value against EC. A 3-miRNA signature (miR-1301-3p, miR-769-5p, and miR-431-5p) has been suggested as a novel prognostic biomarker for EC (7). Li et al. developed a prognostic tool for ESCC based on 8 lncRNA and used weighted gene co-expression network analysis (WGCNA) to evaluate internal interaction between gene expressions (8). A study by Shao et al. identified key genes with potential for use as biomarkers for diagnosis, treatment, and prognosis of ESCC (9).

The immune system has been shown to possess tumor suppressive and tumor promoting properties (10), and cancer immunotherapy has achieved significant success against various malignancies (11). Given that ESCC is characterized by multiple genetic lesions, including a high rate of nonsynonymous mutations and several immunogenic peptides, there is growing interest understanding the benefits of immunotherapy in the context of surgery, chemotherapy, radiation, and other molecular therapies for ESCCs (12). Indeed, many trials are underway to assess immunotherapy as first line treatment in ESCC. Moreover, recent breakthroughs in the use immune checkpoint inhibitors to treat various cancers has encouraged the exploration of their use against ESCC (13). However, not all cancer patients are equally likely to benefit from immunotherapy, highlighting the need to identify which ESCC patients are likely to respond to immunotherapy. Mounting evidence has shown that multiple factors, including immune cell infiltration, interferon (IFN) signaling, deficient DNA mismatch repair, checkpoint expression (PD-1, PD-L1) or high tumor mutational burden affect clinical outcomes (14–16). However, little has been done to characterize the immunogenomic profile in ESCC and to leverage these findings for improved patient outcomes. Additional research is needed to elucidate novel, robust prognostic biomarkers and to personalize ESCC therapy.

Here, we analyzed ESCC datasets obtained from GSE53625. We classified the ESCC patients in GSE53625 into two subtypes based on immunogenomic signature: Immunity High (Immunity_H) and Immunity Low (Immunity_L). Reliability of the classification was verified in the TCGA dataset. We assessed the relationship between subtype and some immune checkpoints, gene expression and clinical features. WGCNA analysis was then used to evaluate the regulatory mechanism of modules related to the Immunity_H group. A concise prognostic signature based on the two immune classes developed and nomogram analysis used to predict the clinical value of the signature *via* integrated bioinformatics approaches. Subsequently, we examined correlation of the signature with drug resistance, immune cell, and immune checkpoint. This risk signature model may be used to predict patient outcomes and improve the precision of ESCC personalized therapy.

## Materials and Methods

### Data Collection and Preprocessing

We downloaded the microarray profiling dataset, GSE53625, as well as the corresponding clinical data from the GEO database. These datasets comprised of 179 ESCC samples and 179 adjacent, matching non-tumor tissues. ESCC mRNA-Seq data for 81 ESCCs, along with associated clinical data, were downloaded from the TCGA database.

### Single-Sample Geneset Enrichment Analysis

The GSE53625 and TCGA cohorts were analyzed to quantify the enrichment level of the twenty-nine immune signatures (17–20)for each ESCC sample using ssGSEA analysis in the R package gsva (21, 22). ESCC patients were grouped into immunity High and immunity Low as per the ssGSEA scores of the twenty-nine immune signatures using hierarchical agglomerative clustering as per Euclidean distance and Ward’s linkage.

### Estimation of Immune Cell Invasion Level, Stromal Content, and Tumor Purity in ESCC

Next, we evaluated the level of immune cell infiltration (immune score), stromal level (stromal score), tumor purity, and estimate score for each ESCC sample using ESTIMATE (23). Results from this analysis were visualized in heat-maps using the pheatmap R package. The correlation between the two subtypes and immune score, estimate score, tumor purity, and the stromal score was analyzed using the ggpubr R package.

### Comparison of the Proportions of Immune Cell Subsets Between ESCC Subtypes

The proportions of 22 human immune cell subsets were calculated using CIBERSORT (24) with the reference of 1,000 permutations and LM22 signature. *P <*0.05 indicated a successful deconvolution standard, indicating that the calculated fractions of immune cell populations were accurate. Results were visualized in heat maps using the pheatmap R package.

### Weighted Gene Co-Expression Network Analysis

WGCNA reveals complex relationships between genes and phenotypes (25). We utilized WGCNA to establish potentially critical gene modules associated with ESCC subtypes using the WGCNA R package. WGCNA analysis involves identification of gene expression similarity-matrix, adjacency matrix as well as the co-expression network. When the correlation between k (the average degree of connectivity) and p (k) reached 0.8, an optimal power value ranging from 1 to 20 was set to build a scale-free topology network. Thus, we set the power value of the soft threshold at 7 to meet the scale-free topology standard. Analysis with a dynamic tree cut algorithm was done to identify gene co-expression modules. The relevance between modules and subtypes was analyzed to determine the related module.

### Establishment of Differentially Expressed Immune-Related Genes and Construction of Training Set and Testing Set

We analyzed for the differentially expressed genes (DEGs) between tumor samples and normal samples in the GSE53625 dataset using the limma R package to determine the differentially expressed immune-related genes (DE IRGs) associated with ESCC prognosis. Similarly, we identified the immune-related genes (IRGs) differentially expressed between the Immunity High and Immunity Low groups. The DEGs and IRGs with an adjusted *P* <.05 as well as absolute log2-fold change (FC) >1 were selected for further analyses. We identified the DE IRGs at the points of intersection between the DEG and the IRG lists. Because of the limited number of normal esophageal tissue in the TCGA database, we split the GSE53625 dataset randomly into training as well as the testing sets (7:3). We used the training set to construct a potential prognostic signature and the testing for validation.

### Establishment and Validation of an Immune Prognostic Predictive Signature

We used the Univariate Cox proportional hazards regression analysis to identify DE IRGs capable of predicting overall survival (OS). A log-rank test analysis was done, and a *P* <.05 threshold was set to identify the candidate genes. Next, we performed the least absolute shrinkage and selection operator (LASSO) Cox (26) examination to identify more relevant IRGs. LASSO Cox regression analysis was done by performing 1000 substitution samplings in the dataset and genes with a recurrence frequency greater than 900 selected. Next, we used the multivariate Cox regression investigation of these genes to determine those with the best prognostic value and construct the prognostic signature. The prognosis index (PI) = (βmRNA1* expression level of mRNA1) + (βmRNA2* expression level of mRNA2) +…+ (βmRNAn* expression level of mRNAn) and the β acquired from multivariate Cox regression analysis. We computed the risk score of each patient and classified them into the low-risk or high-risk group as per the median risk score of the training database as the cutoff. We further used the Kaplan-Meier survival examination and the time-dependent receptor working characteristic (ROC) curve analysis to assess the predictive value of the prognostic signature. Finally, we validated the signature using the testing cohort.

### Independence of the Immune Prognostic Signature and Construction of a Predictive Nomogram

Univariate and multivariate Cox proportional hazard regression investigations were done to assess whether the immune prognostic signature has independent prognostic value. Next, a nomogram (27) was built using all the independent prognostic factors to predict the prognostic value at 1-,3-, and 5-year survival of ESCC patients. We then plotted calibration plots for internal validation. We used the ROC curve examination to compare the predictive performance of single independent prognostic factors to the nomogram. After that, we performed a decision curve analysis (DCA) to assess the clinical net benefit (28).

### GDSC and TIMER Database Analysis

Next, the drug response data (defined by IC50 value), as well as the gene bulk expression profiles of cancer cell lines, were downloaded from the GDSC (Genomics of Drug Sensitivity in Cancer) database. GDSC constitutes the most extensive public arsenal of data on drug sensitivity in cancer cells as well as molecular markers of drug response (29). Next, we predicted the IC50 value for each drug using the LIBSVM package in R under default parameters and linear kernel. We then compared the differences between the low-risk and high-risk groups to establish whether the two groups exhibit different drug sensitivities. We then evaluated the association between abundant tumor immune infiltrates (CD4+ T-cells, CD8+ T-cells, B-cell, dendritic cells, macrophages, and neutrophils) as well as the expression levels of the selected genes using the TIMER (Tumor Immune Estimation Resource) platform. TIMER is used to explore and visualize immune infiltrates comprehensively among different types of cancer (30, 31). Purity-corrected partial Pearson’s correlation and its statistical significance were visualized using correlation graphics.

### Statistical Analysis

Wilcox test was used in comparing the differences between groups. We used the Database for Annotation, Visualization, and Integrated Discovery to perform the GO term analysis of biological processes (GO_BP) and KEGG analysis of pathway enrichment. Statistical analyses were performed using the R software.

## Results

### ESCC Patients Cluster Into Immunity High and Immunity Low Groups

**Figure 1:**
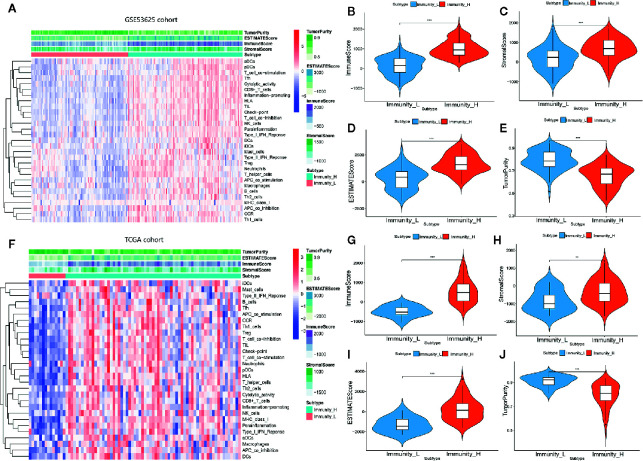
Hierarchical clustering of ESCC yielding two stable subtypes in two different datasets. **(A)** Heat map of ESCC clustering in GSE53625 database. **(B–E)** Comparison of the Immune Score, Estimate Score, Stromal Score, and Tumor purity in GSE53625 database. **(F)** Heat map of ESCC clustering in TCGA database. **(G–J)** Comparison of the Immune Score, Estimate Score, Tumor purity, and Stromal Score in TCGA database. *P < .05, **P < .01, ***P < .001.

We examined 29 immune-related gene sets representing multiple immune cell types, pathways, and functions (**Supplementary Table 1**). The enrichment levels of immune cells, pathways, and functions were quantified using the ssGSEA assessment of the ESCC samples. ssGSEA assessment of the 29 gene signature was used to hierarchically cluster the ESCC cases in the GSE53625 dataset, with two distinct clusters emerging (**Figure 1A**). Evaluation of the TCGA database revealed identical clustering (**Figure 1F**). The two clusters were designated as Immunity High (Immunity_H) and Immunity Low (Immunity_L). Since tumors consist mainly of tumor cells, immune cells, and stromal cells, we analyzed for the immune cell infiltration levels (immune scores), stromal cell content (stromal scores), and tumor purity between the two groups. Consequently, we found that immune scores, stromal scores as well as the estimate scores were markedly higher in Immunity_H cluster than in the Immunity_L arm in both datasets. However, we reported a contrasting trend for tumor purity (**Figures 1B–E, G–J**). These findings indicated that Immunity_H patients possess more immune and stromal cells compared to Immunity_L patients, while Immunity_L patients have more tumor cells.

### HLA and Hot Immune Checkpoint Genes Are Overexpressed in Immunity High Patients

**Figure 2:**
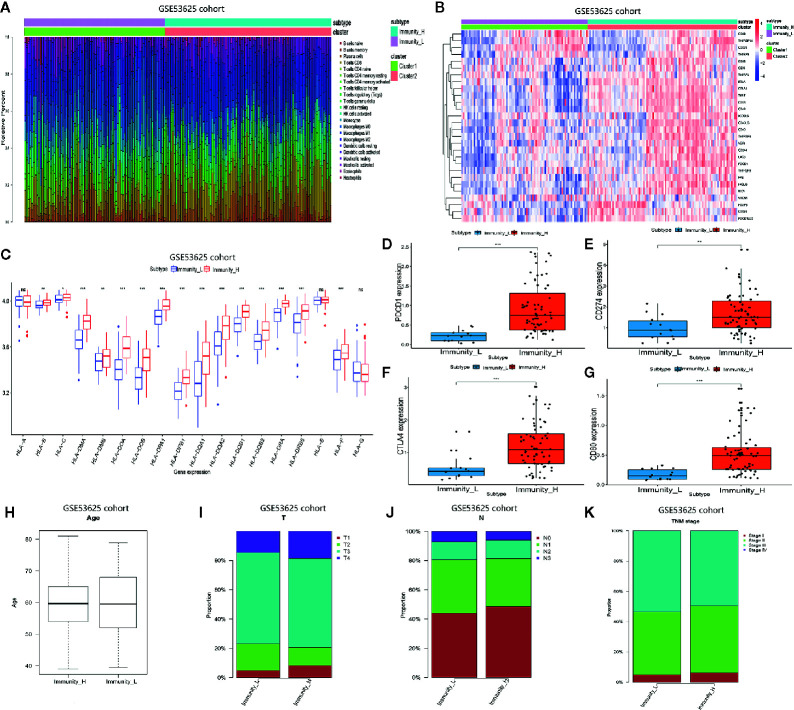
Two ESCC subtypes showing different phenotype in GSE53625 database. **(A)** Proportion of the immune cell invasion between ESCC subtypes. **(B)** Heat map of the expression levels of different gene between ESCC subtypes. **(C)** Comparison of the expression of HLA genes between ESCC subtypes. **(D–G)** Comparison of PDCD1, CD274, CTLA4, and CD80 expression levels between TNBC subtypes. **(H–K)** The relationship between ESCC subtypes and clinical characteristics including age as well as the TNM stage. *P <.05, **P <.01, ***P <.001.

**Figure 3:**
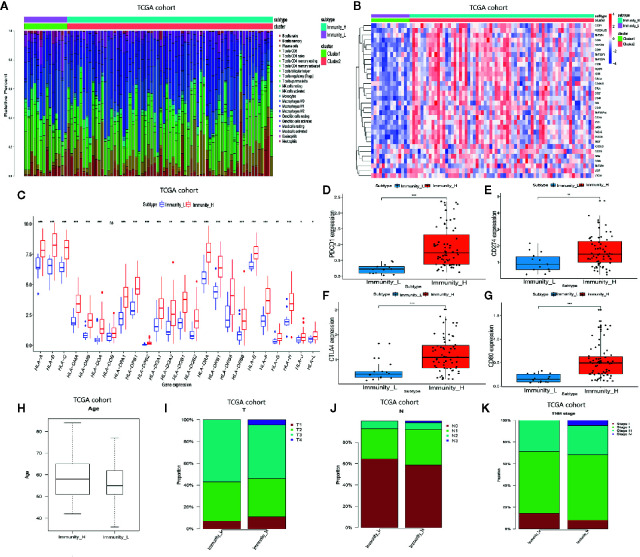
Two ESCC subtypes showing different phenotype in TCGA database. **(A)** Proportion of the immune cell invasion between ESCC subtypes. **(B)** Heat map of the expression profiles of different genes between ESCC subtypes. **(C)** Comparison of the expression levels of HLA genes between ESCC subtypes. **(D–G)** Comparison of PDCD1, CD274, CTLA4, and CD80 expression levels between TNBC subtypes. **(H–K)** The relationship between ESCC subtypes and clinical characteristics including age as well as the TNM stage. *P <.05, **P <.01, ***P <.001.

We analyzed the GSE53625 dataset and established the proportions of 22 immune cell subsets using CIBERSORT as well as the expression level of some genes in the two clusters (**Figures 2A, B** **)**. The expression of most HLA genes in the Immunity_H arm was significantly higher relative to the Immunity_L group (**Figure 2C**). Moreover, we examined the expression levels of some hot immune checkpoints in the two groups, including PDCD1, CD274, CTLA4, and CD80. The results indicated that the expression of these genes was remarkably higher in the Immunity_H arm relative to the Immunity_L group (**Figures 2D–G**). These results suggested that the Immunity High ESCC patients responded better to the immune checkpoint inhibitors than the Immunity Low ESCC patients since immune checkpoint expression is positively associated with the immunotherapeutic effect. Next, we assessed the relationship between the two subtypes and clinical features (**Figures 2H–K**). Finally, these findings were verified using the TCGA dataset. The distribution of the immune cell subsets and expression analysis of some genes in the two subtypes are shown in **Figures 3A, B**. The expression levels of most HLA genes, PDCD1, CD274, CTLA4, and CD80 in the Immunity_H group were also higher than in the Immunity_L arm, consistent with the above results (**Figures 3C–G**). The relationship between the two subtypes and clinical characteristics is shown in **Figures 3H–K**.

### Gene Modules Are Significantly Associated With Immunity High

**Figure 4:**
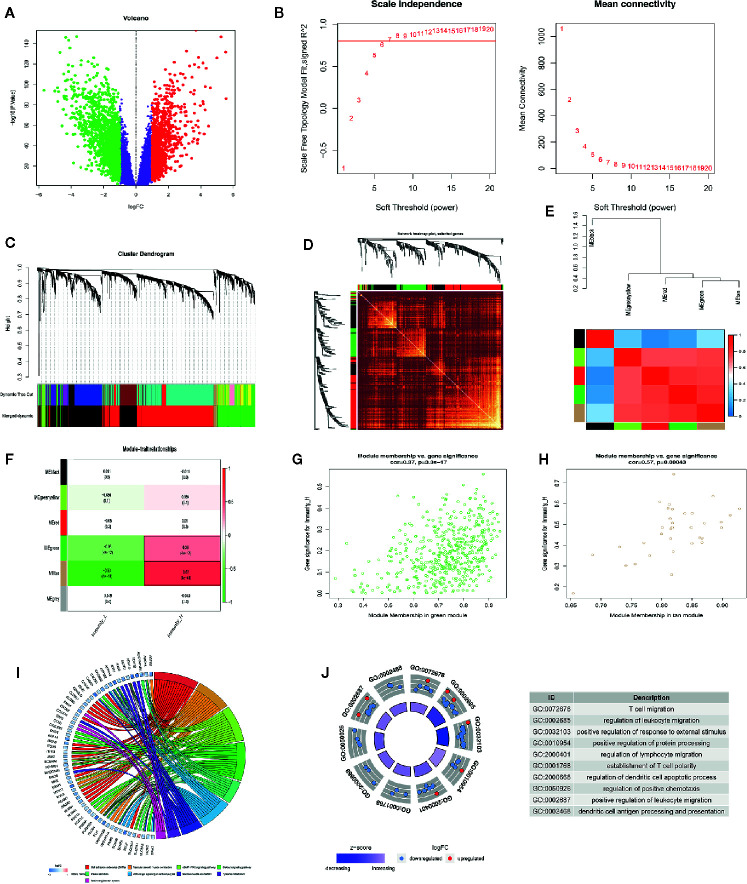
WGCNA analysis indicating ESCC subtypes-specific pathways, gene ontology. **(A)** Volcano plot indicating differentially expressed genes between ESCC and normal tissues. **(B)** Investigating scale-free topology model fit measure of different soft-thresholding powers (β) as well as the mean connectivity of various soft-thresholding powers. **(C)** Clustering gene dendrogram based on Dynamic Tree Cut algorithm. **(D)** Analyzing the connectivity of critical genes in different module. **(E)** Hierarchical clustering as well as the heat map of the hub gene network. **(F)** Heat map showing the relationship between ESCC subtypes and module eigengenes. **(G, H)** Scatter plot showing module eigengenes in green and tan modules. **(I, J)** KEGG pathway and GO enrichment of genes in green and tan modules.

We performed a WGCNA analysis to identify the gene modules associated with the ESCC subtypes. First, the DEG analysis between tumor and normal groups in the GSE53625 database was done (**Figure 4A**). At a soft threshold power (β) of 7, the association between genes reached a scale-free network distribution (**Figure 4B**). Next, we used a dynamic tree-cutting algorithm to identify 6 distinct co-expression modules with different numbers of genes (**Figure 4C**). The grey module consisted of a gene set that was not designated to any of the modules. Connectivity examination of the crucial genes in discrete modules is shown in **Figure 4D**. Notably, we found a strong association between the green and tan modules (**Figure 4E**). Analysis of the linear mixed-effects model revealed that the tan module genes (t-value=0.63, *P*=4e-41) and the green module genes (t-value=0.36, *P*=4e-12) are markedly associated with the Immunity High group (**Figure 4F**). A scatter plot of multiple module memberships for each gene contained in these modules revealed similar findings (**Figures 4G, H** **)**. We next performed GO term and KEGG pathway analysis of the genes in the green and tan modules to understand their roles. The KEGG analysis revealed that the genes related to the two modules are involved in the following pathways; cell adhesion molecules (CAMs), vascular smooth muscle contraction, cGMP−PKG signaling pathway, adrenergic signaling in cardiomyocytes, and the calcium signaling pathway (**Figure 4I**). GO term analysis revealed that the genes significantly regulate immune response processes (including T cell migration) and regulation of protein processing (**Figure 4J**).

### The Immune-Related Prognostic Signature Has a Good Prognostic Performance

**Figure 5:**
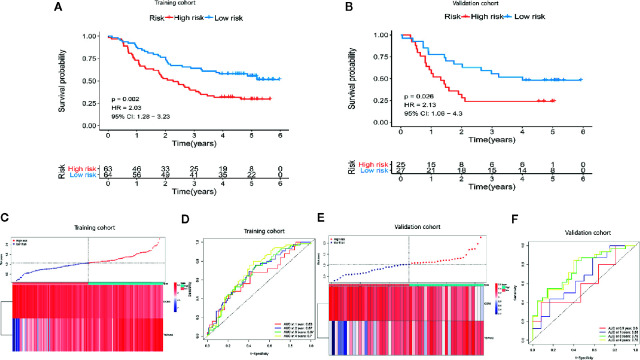
Establishment of a two-gene immune prognosis signature. **(A, B)** Kaplan-Meier survival curves designating OS among the risk stratification clusters in the training and validation cohort**. (C, E)** Distribution of risk scores of ESCC patients with different gene expression levels in the training as well as the validation cohort. **(D, F)** Time-dependent ROC analysis with calculated AUCs for OS prediction in the training as well as the validation cohort.

We compared gene expression data between ESCC tissues and adjacent non-tumor tissue in GSE53625 and identified the DEGs (|LogFC| >1, *P* <.05). We used the same method to identify DE IRGs expressed in the Immunity_H and Immunity_L classes. Consequently, we identified 662 DE IRGs, which we subjected to further analysis (**Figure S1**). Next, 127 genes that significantly correlated with ESCC OS were identified using univariate Cox regression analysis (*P*=<.01). After that, the LASSO Cox analysis was performed to reduce further the number of candidate genes with the penalty parameter set at 10-fold cross-validation. Therefore, 6 genes that emerged more than 900 times following a 1,000 repetition were selected (**Figure S2**). Finally, we carried out a multivariate Cox regression analysis and identified two genes (CCR5 and TSPAN2). These two genes were used to construct a prognostic signature, the prognostic index (PI) = (-0.288 * expression level of CCR5) + (0.176 * expression level of TSPAN2). The risk score for each patient was then calculated, and the patients classified into high-risk or low-risk groups based on the median risk score as the optimal cutoff point. Kaplan-Meier survival analysis revealed that the OS in the high-risk group was significantly lower than in the low-risk group (*P*=0.002, HR = 2.03) (**Figure 5A**). Time-dependent ROC curve analysis showed the predictive value of the signature (**Figures 5C, D** **)**. The area under the time-dependent ROC curves (AUCs) for 1-, 2-, 3-, and 4-year OS were 0.63, 0.67, 0.67, and 0.7, respectively, indicating our prognostic signature has good performance. The performance was verified using the validation cohort. Based on these findings, ESCC patients were grouped into high-risk and low-risk groups. Patients in the high-risk group exhibited dismal survival relative to those in the low-risk group (*P*=0.026, HR = 2.13) (**Figure 5B**). A great difference in risk-score distribution and gene expression was observed (**Figure 5E**). The AUCs of the two-gene prognostic signature were 0.6, 0.65, 0.76, and 0.76 for the 0.5-, 1-, 3-, and 4-year survival, respectively (**Figure 5F**). Therefore, our prognostic model demonstrated a high degree of sensitivity and specificity.

### Combining Our Prognostic Signature With the TNM Stage Enhances Prognostic Power

**Figure 6:**
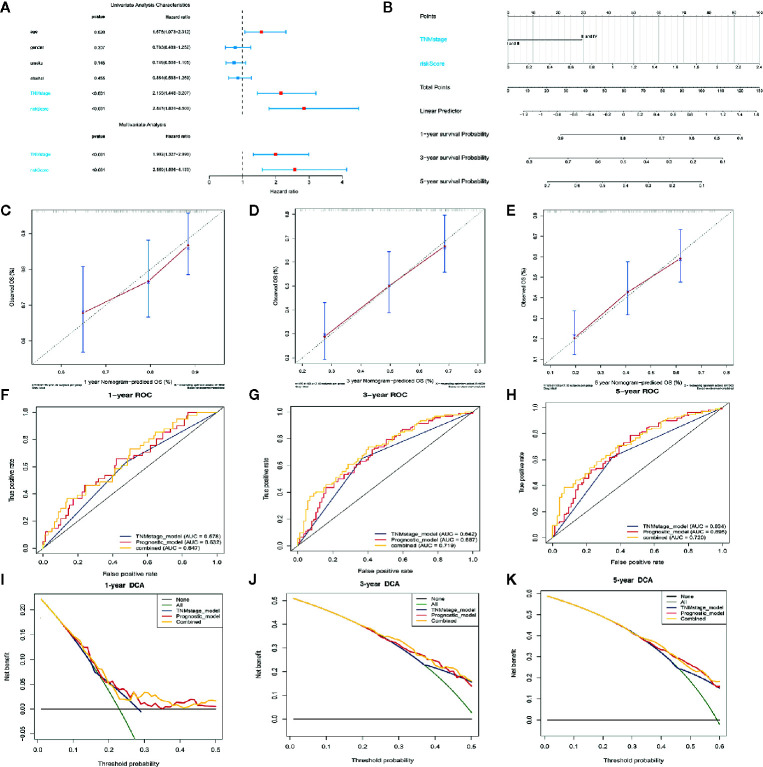
Construction of the nomogram model. **(A)** Forrest plot showing the univariate as well as multivariate analyses indicating association of the prognostic model and clinicopathological features with overall survival. **(B)** The nomogram was constructed as per two independent prognostic factors for predicting OS in ESCC patients at 1, 3, and 5 years. **(C–E)** Nomogram calibration plots for predicting the probability of OS at 1, 3, and 5 years. **(F–H)** Nomogram time-dependent ROC curves for 1-,3-, and 5-year OS. **(I–K)** Nomogram DCA curves for 1-,3-, and 5-year OS to evaluate the clinical decision-making benefits of the nomogram.

We used univariate and multivariate Cox regression analyses to evaluate the independence of our prognostic model using standard prognostic indicators. The results demonstrated that the TNM stage (*P*= <.001, HR = 1.992) and the risk score (*P*=<.001, HR = 2.596) were independent indicators of OS (**Figure 6A**). Predictive nomogram analysis based on the two independent prognosis factors was used to evaluate the clinical prognosis. Each independent prognosis factor was assigned a score, and the total point value obtained by summing the respective scores corresponding to each prognostic variable. The corresponding OS for a patient at 1, 3, and 5 years was determined (**Figure 6B**). We performed an internal validation of the nomogram using the calibration plot that indicated consistency between predicted OS outcomes and actual observations (**Figures 6C–E**). The C index of the nomogram in OS prediction was 0.65, which was superior to the C index of the TNM stage (0.59) and the prognostic signature (0.64). These findings indicated that our model has a higher prediction power. By combining our prognostic model with the TNM stage, the AUCs for 1-, 3-, and 5-year OS were 0.647, 0.720, and 0.719, respectively, which were better than models relying on single independent predictive factors (**Figures 6F–H**). Next, we used DCA to evaluate the suitability of this model in clinical settings and established that the combined model is the best for predicting OS (**Figures 6I–K**).

### High-Risk ESCC Patients Exhibit Resistance to Paclitaxel, Gefitinib, and Erlotinib et Cancer Drugs

**Figure 7:**
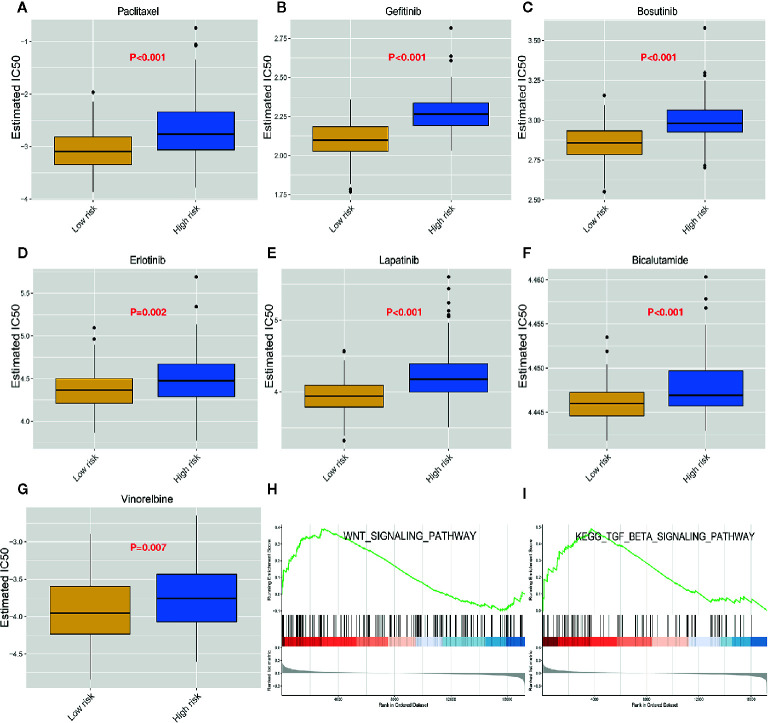
Heterogeneous drug resistance and gene set enrichment analysis in the low-risk and high-risk score groups. **(A–G)** Comparison of the computed IC50 between the high-risk and low-risk groups of paclitaxel, Gefitinib, Bosutinib, Erlotinib, Lapatinib, Bicalutamide, and Vinorelbine. **(H, I)** Gene set enrichment analysis between the high-risk and low-risk groups.

Next, we downloaded data on the response of cancer cells to various drugs from the GDSC database. To predict the IC50 for each drug, we analyzed drug response data (IC50) and robust multi-array (RMA) gene expression profiles obtained from the GDSC database. IC50s helps to quantify the ability of a drug to induce apoptosis, which is inversely related to drug sensitivity. We compared the estimated IC50s of some drugs in the low-risk and high-risk ESCC subtypes (**Figures 7A–G**). Paclitaxel, gefitinib, and erlotinib are commonly used drugs to treat ESCC. Our analysis revealed that the IC50s of these drugs were significantly higher in the high-risk than in the low-risk ESCC subtypes. This suggests that high-risk ESCC patients cannot benefit from treatment using these drugs due to drug resistance. Moreover, GSEA analysis of the biological functions in the immune-related model, revealed that the genes highly expressed in the high-risk group are significantly enriched in various signaling pathways, including WNT and TGF-β signaling pathways (**Figures 7H, I** **)**. The impaired TGF-β signaling pathway is associated with inflammatory disorders, tumorigenesis, and immunosuppression in the tumor microenvironment (32). Further studies on the relationship between the signature and immune cell infiltration should be conducted.

### Immune Infiltration Cells Are Positively Associated With the Prognostic Signature

**Figure 8:**
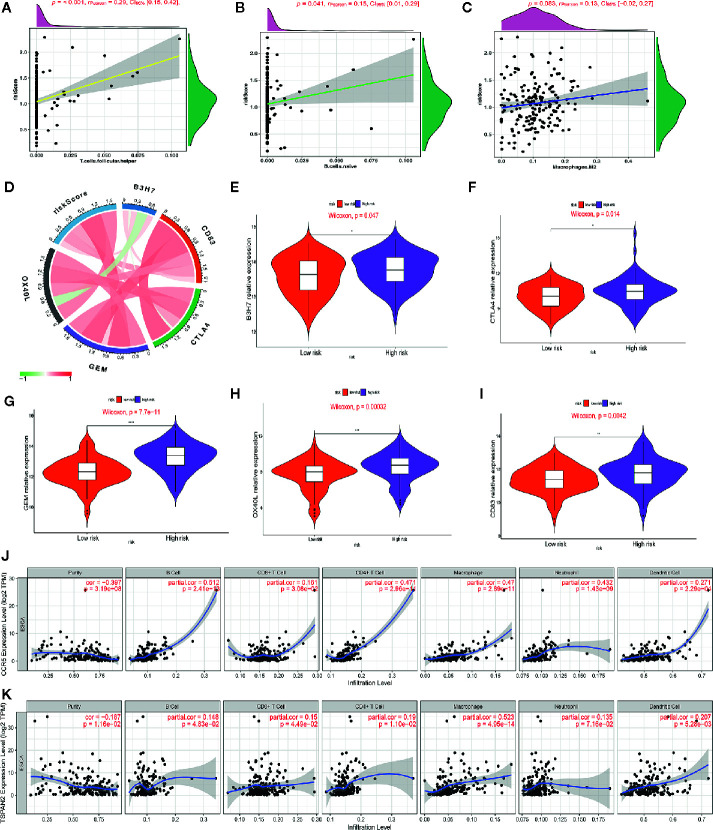
Association of the risk score with immune cell infiltrating levels and immune checkpoints. **(A–C)** Correlation analysis between infiltrating level of follicle-helper T cells, naive B cells and M2 macrophages and risk score. **(D)** The relationship between the risk score and the expression of several critical immune checkpoints. **(E–I)** Violin plots visualizing markedly different immune checkpoint expression levels between the high-risk and low-risk patients. **(J, K)** Partial correlation analysis between genes expression (CCR5, TSPAN2) and the level of tumor immune infiltrates (CD4+ T-cells, CD8+ T-cells, B-cell, macrophages, dendritic cells, and neutrophils) in TIMER database.

We studied the immune microenvironment status by evaluating the features of immune infiltration that favor tumor-immune interaction. This analysis revealed that tumor-infiltrating immune cells, including T helper cells, naive B cells, and M2 macrophages, were positively associated with our signature (**Figures 8A–C**). There has been increasing interest in immune checkpoints due to their essential roles in immune modulation, and immune checkpoint inhibitors have potential application in cancer treatment. Therefore, we assessed the association between the signature and the expression of immune checkpoint modulators, including B3H7, CTLA4, CD83, OX40L, and GEM. Consequently, the risk score was positively associated with the expression of these genes (**Figure 8D**). Additionally, we compared the expression of B3H7, CTLA4, CD83, OX40L, and GEM in low-risk and high-risk ESCCs. Expressions of these genes were remarkably higher in the high-risk ESCC group relative to the low-risk HCC group. This suggests that the dismal prognosis exhibited by high-risk ESCC patients is at least partially caused by the immunosuppressive microenvironment (**Figures 8E–I**). Finally, we used TIMER database to study the link between abundant tumor immune infiltrates (CD4+ T-cells, CD8+ T-cells, B-cell, neutrophils, macrophages, and dendritic cells) and the expression of CCR5 and TSPAN2. Our results showed that the expression level of CCR5 was linked to the presence of B cells (part.cor=0.512), CD4+T cells (part.cor=0.471), macrophages (part.cor=0.47) and neutrophils (part.cor=0.432) (**Figure 8J**). The expression level of TSPAN2 was associated with infiltration by B cells (part.cor=0.148), CD4+T cells (part.cor=0.19) and macrophages (part.cor=0.523) (**Figure 8K**). These results independently validated the connection between our signature and infiltrating immune cells.

## Discussion

Esophageal squamous cell carcinoma (ESCC) constitutes the most common esophageal cancer (EC) subtype among African and Asian populations. ESCC is associated with >400,000 deaths annually (33–36). Presently, pathological analysis of various cancer types does not take into account tumor heterogeneity at the molecular and genetic levels (37, 38). Owing to this heterogeneity, patients at the same cancer stage could have completely different clinical outcomes after receiving a similar therapy. The recent advances in data analysis and high-throughput sequencing enable further research into the molecular heterogeneity of ESCC to develop personalized ESCC treatment.

A few previous studies have stratified subtypes of ESCC based on genomic profiling (7–9, 39). However, the stratification of ESCC by immune signatures is poorly studied. Herein, we classified ESCC patients into two distinct subtypes based on immunogenomic profiling comprising of the Immunity High and Immunity low profiles. Furthermore, our analysis showed that this classification is reproducible and predictable. Immune scores were considerably higher in the Immunity High profile suggesting higher immune activity in Immunity High ESCC patients. Our findings revealed that significantly higher levels of most human leukocyte antigen (HLA) genes are expressed in the Immunity High ESCC subtype, indicating more potent immunogenicity compared to the Immunity Low subtype. Immunotherapy has been extensively studied (40), and immune checkpoint inhibitors show potential applications in several refractory cancers in therapeutic development (41–43). However, less than 20% of cancer patients have benefited from immunotherapy (18). Therefore, ESCC classification using an immune signature could help in the identification of patients who could well respond to immunotherapy. Due to the stronger immunogenicity, Immunity High ESCC patients are more likely to respond to immunotherapy. Such patients are more likely to benefit from immune checkpoint inhibitors, since PD-L1, PD-1, CTLA4, and CD80 are highly expressed in Immunity High ESCC patients. WGCNA analysis of the underlying regulatory mechanism of modules related to the Immunity High group revealed that the genes in the associated modules are significantly enriched in immune responses and several cancer-associated pathways, including cell adhesion molecules (CAMs), vascular smooth muscle contraction, the calcium signaling pathway, and the cGMP−PKG signaling pathway. The relationship between these pathways and immunity in ESCC requires further investigations.

We exploited high-throughput data analysis methods and databases to elucidate novel ESCC prognostic biomarkers and constructed an immune-related prognostic signature based on CCR5 and TSPAN2. CCR5 (C-C chemokine receptor type 5) is expressed in T cells, other leukocytes, macrophages, and certain types of cancer cells (44). This gene plays an important role in recruiting leukocytes into target sites (44, 45). The interaction between CCR5 and its ligand (CCL5) drives cell proliferation, immunosuppression, angiogenesis, and migration, thereby promoting tumorigenesis (44, 46–48). TSPAN2 is a member of a transmembrane-spanning protein family, which removes intracellular reactive oxygen species through CD44-mediated pathways; thus, enhancing cell motility and invasiveness (49). Elevated TSPAN2 levels are associated with dismal prognosis in lung cancer (49, 50). Our results revealed that this two-gene signature has independent prognostic value in ESCC patients. We developed a robust nomogram model that offers excellent prognosis capacity by integrating the corresponding value of molecular and clinical features. However, resistance to chemotherapy, which limits long-term cancer patient outcomes, remains an important challenge in oncology. Recent advancements in targeted anti-cancer therapies is a breakthrough. However, even after an initial response, various cancer types develop resistance to targeted therapy (51). The mechanisms of drug resistance are complex, with no existing approaches to accurately predicting their effectiveness. Herein, we predicted the immune signature using 266 chemotherapeutic and targeted drugs on the GDSC database. Our results showed that the immune signature is associated with resistance to chemotherapy and targeted drugs, including paclitaxel, gefitinib, and erlotinib, providing a method of personalizing treatment and limiting resistance. The GSEA analysis results revealed that our immune signature promotes drug resistance by regulating the TGF-β signaling pathway.

The tumor microenvironment (TME) is thought to enable cancer cell immune evasion, inhibit apoptosis, and promote proliferation, angiogenesis, invasion, and metastasis (52). Studying immune infiltration is essential to elucidate the relationship between tumors and immunity. Herein, we evaluated the association between the immune signature and immune cell infiltration to elucidate the immune microenvironment status in ESCCs. The immune signature was found to be positively associated with tumor infiltration through immune cells, including T helper cells, naive B cells, and M2 macrophages, which was validated by analysis of the TIMER database. This implies that the heterogeneity of immune infiltration is essential for ESCC development. Thus, this signature predicts high immune cell infiltration, which has important clinical implications. Analysis of the relationship between risk score and the expression of crucial immune checkpoint genes revealed that high-risk patients had higher CTLA4, CD83, B3H7, OX40L, and GEM levels in the tumor microenvironment. This implies poorer outcomes for these patients are at least partially caused by an immunosuppressive microenvironment, and these patients respond better to immune checkpoint inhibitors.

A limitation of this study is that crucial modulators of ESCC prognosis could have been missed when adjusting for the weight of the regression coefficient in LASSO. Secondly, our signature has only been validated internally. The retrospective nature of this study calls for further validation using a prospective investigation.

## Conclusion

Herein, we categorized ESCC patients into two classes with latent clinical implications for ESCC treatment based on immune signatures and constructed a two-gene immune prognostic model. The proposed immune prognostic model has the potential to predict ESCC outcomes and guide personalized therapy.

## Data Availability Statement

Publicly available datasets were analyzed in this study. This data can be found here: https://www.ncbi.nlm.nih.gov/geo/ http://cancergenome.nih.gov/.

## Author Contributions

JG and GL contributed to conception and design of the study. TT and BZ organized the database. JG performed and wrote the manuscript. All authors contributed to the article and approved the submitted version.

## Conflict of Interest

The authors declare that the research was conducted in the absence of any commercial or financial relationships that could be construed as a potential conflict of interest.

## Supplementary Material

Supplementary Figure 1 The Venn diagram showing the overlapped genes between DEGs and IRGs lists.

Supplementary Figure 2 LASSO analysis to screen out candidate genes.
