# Supplementary figures and images for: A Prognostic Signature Based on Immunogenomic Profiling Offers Guidance for Esophageal Squamous Cell Cancer Treatment
Source: Front Oncol. 2021 Feb 24;11:603634. doi: 10.3389/fonc.2021.603634 (PMC7943886; doi:10.3389/fonc.2021.603634)

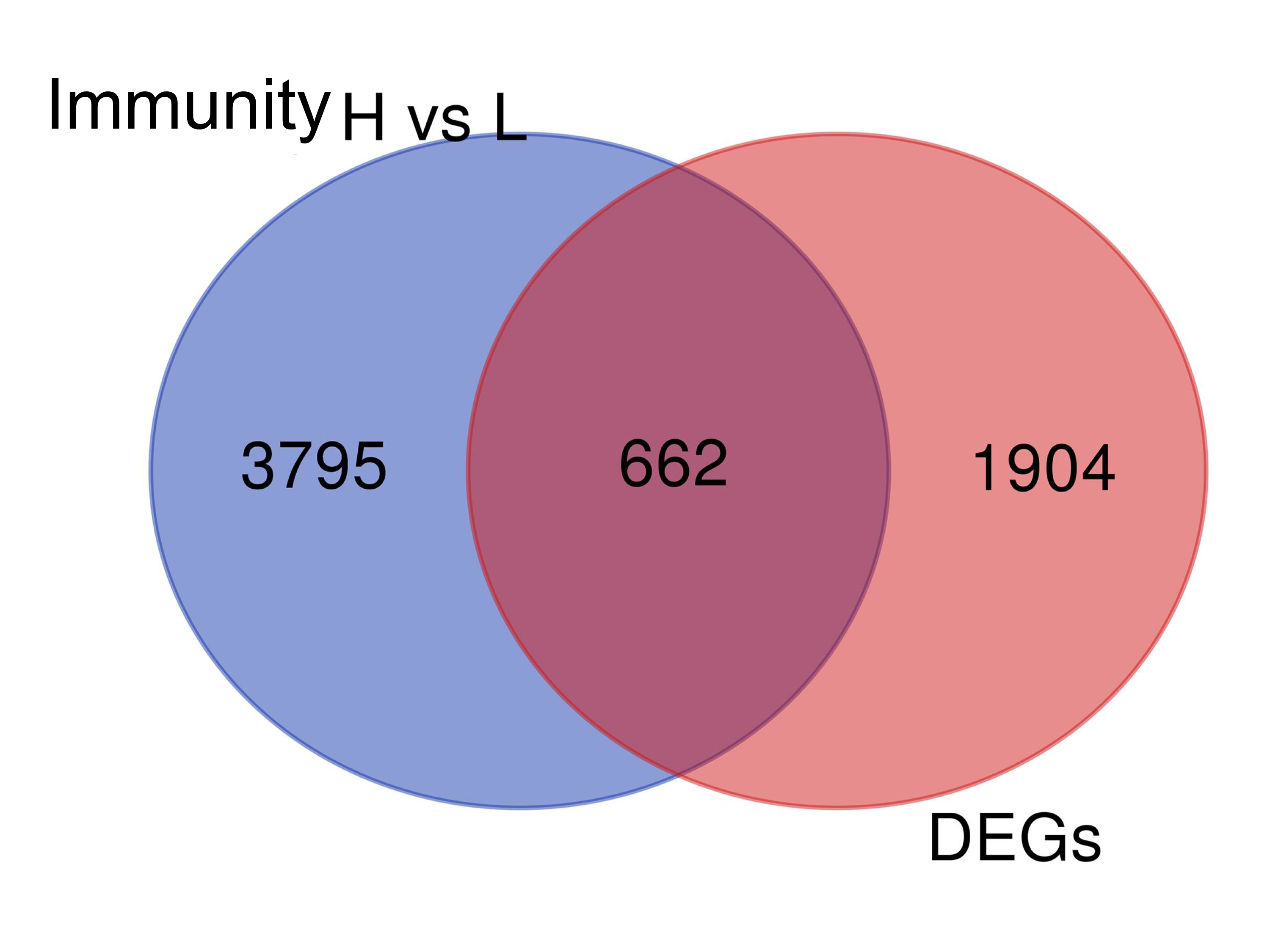

Supplement: Supplementary Figure 1 — The Venn diagram showing the overlapped genes between DEGs and IRGs lists. [file Image_1.tif]

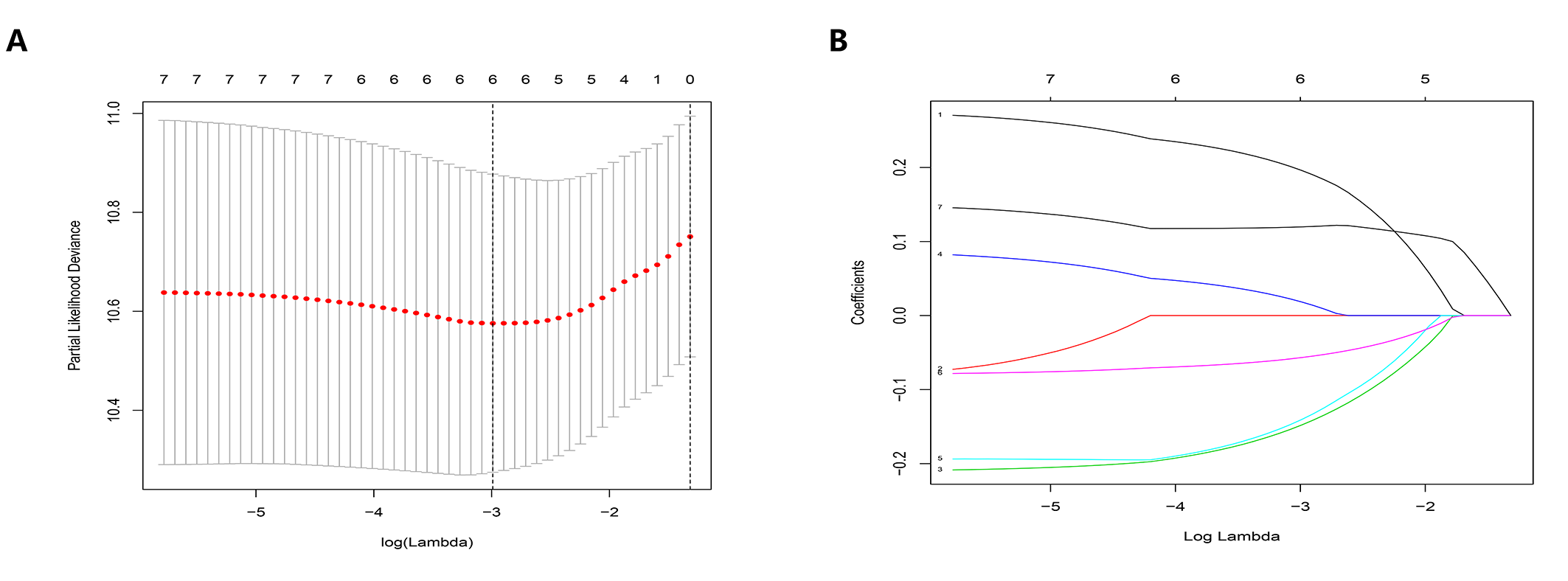

Supplement: Supplementary Figure 2 — LASSO analysis to screen out candidate genes. [file Image_2.tif]
